# Dietary modification and nutritional supplements for exercise-induced bronchoconstriction: a systematic review and meta-analysis

**DOI:** 10.3389/fphys.2026.1975449

**Published:** 2026-10-13

**Authors:** Mingxin Su, Richen Sun, Changkun Shan

**Affiliations:** 1 College of Physical Education, Central China Normal University, Wuhan, China; 2 School of Sports Training, Guangzhou Sport University, Guangzhou, China; 3 Department of Public Health, Jinan Vocational College of Nursing, Jinan, China

**Keywords:** dietary modification, exercise-induced asthma, exercise-induced bronchoconstriction, forced expiratory volume in 1 second, meta-analysis, nutritional supplementation, systematic review

## Abstract

**Objective:**

Our aim was to evaluate the effects of dietary modification and nutritional supplementation on exercise-induced bronchoconstriction (EIB) and the certainty of evidence for each intervention.

**Methods:**

PubMed, Web of Science, Scopus, and CENTRAL were searched through to 1 August 2026. Randomized parallel-group and crossover trials assessing EIB with an exercise challenge, eucapnic voluntary hyperpnea (EVH), or another indirect bronchial provocation test were included. The primary outcome was the maximum or peak percentage fall in forced expiratory volume in 1 s (FEV_1_) within 30 min, with fixed-time-point values treated as proxy outcomes. Comparable intervention-specific effects were synthesized, crossover trials were analyzed as paired designs, and Risk of Bias 2 (RoB 2) and Grading of Recommendations Assessment, Development, and Evaluation (GRADE) were applied.

**Results:**

A total of 20 studies (22 reports) were included. Low sodium compared with experimentally prescribed high sodium, standard omega-3 fatty acids/fish oil, selected caffeine doses, and PCSO-524 showed potentially favorable signals. However, the evidence was limited by the small sample sizes, proxy outcomes, heterogeneity, risk of bias, missing paired data, or lack of independent replication. Adverse events were inadequately reported.

**Conclusion:**

Current evidence is insufficient to recommend any nutritional intervention for routine prevention or treatment of EIB. Nutritional approaches should not replace guideline-based assessment or pharmacological management.

**Systematic review registration:**

https://www.crd.york.ac.uk/PROSPERO/, identifier CRD420261485221.

## 1 Introduction

Exercise-induced bronchoconstriction (EIB) is a transient, reversible airway narrowing induced by exercise or hyperventilation, which can occur in patients with or without underlying asthma (Parsons et al., 2013; Hostrup et al., 2024). The symptoms lack specificity and cannot be diagnosed solely based on cough, wheeze, or chest tightness and should be confirmed based on objective lung function changes after an exercise stress test, eucapnic voluntary hyperpnea (EVH), or other indirect bronchial provocation tests (Parsons et al., 2013; Weiler et al., 2016; Koya et al., 2020). The maximum percentage decrease in forced expiratory volume in 1 s (FEV_1_) relative to the baseline within 30 min after exercise is a commonly used core indicator (Parsons et al., 2013; Anderson and Kippelen, 2023).

EIB involves processes such as loss of airway water and heat, changes in the osmotic load, and the release of inflammatory mediators, ultimately leading to reversible airflow limitation (Kippelen et al., 2018; Anderson and Kippelen, 2023). For patients with asthma, standard inhaled anti-inflammatory treatment and preventive medication before exercise are still the basis of management. Nutritional intervention can only be used as a possible auxiliary measure and cannot replace standard treatment (Parsons et al., 2013; Hostrup et al., 2024; Global Initiative for Asthma, 2026).

EIB should also be interpreted according to the clinical context. In patients with established asthma, it commonly occurs within a chronic inflammatory airway disease, and management begins with optimization of asthma control. In athletes or physically active individuals without otherwise diagnosed asthma, repeated high ventilation and exposure to cold or dry air, pollutants, or irritants may contribute to a different phenotype and treatment history. These groups may therefore differ in background therapy, airway inflammation, and the relevance of a nutritional intervention. Objective confirmation remains essential in both settings (Kippelen et al., 2018; Koya et al., 2020; Hostrup et al., 2024).

A low-sodium diet, omega-3 polyunsaturated fatty acids, antioxidant nutrients, caffeine, and microbiota-related supplements may affect EIB-related inflammation, oxidative stress, or airway reactivity (Marathe et al., 2022; O'Brien et al., 2022; Mokra et al., 2023; Ogbu et al., 2023; Patchen et al., 2023; Rodrigues et al., 2023; Song et al., 2023; Zajac and Wojciechowski, 2023; Zhao et al., 2023; Perdijk et al., 2024; Reddy et al., 2024; Sogard et al., 2025). However, studies vary significantly in formulation, dosage, duration, comparison conditions, and populations, and observational evidence of the overall dietary patterns cannot replace randomized trial evidence of EIB (Rodrigues et al., 2023; Bao et al., 2025).

The proposed mechanisms differ by intervention. Exercise hyperpnea increases airway water loss and airway surface osmolarity, which can trigger inflammatory mediator release and bronchial smooth muscle contraction. Sodium and chloride intake may modify this osmotic response and airway reactivity, omega-3 fatty acids may alter eicosanoid production, antioxidants may limit the reactive oxygen species generated during airway stress, and caffeine may provide short-term bronchodilation through adenosine receptor antagonism and related methylxanthine effects. Microbiota-directed supplements may influence immune signaling through the gut–lung axis. These mechanisms justify investigation, but do not establish clinical efficacy (Marathe et al., 2022; Patchen et al., 2023; Song et al., 2023; Sogard et al., 2025).

Most previous evidence syntheses were narrative reviews, and crossover trials with small sample sizes were common, and crossover trials with small sample sizes were common. If the within-subject pairing structure is ignored, the accuracy of the effect may be misestimated. If nutritional interventions with different mechanisms and clinical positioning are combined into a single “overall nutritional effect,” clinical interpretability is lacking (Higgins et al., 2024b; Higgins et al., 2024a; Sogard et al., 2025). In addition, the objective diagnosis, athlete status, and background treatment of EIB are inconsistent, limiting extrapolation (McKay et al., 2022; Wilkins et al., 2025).

Unlike the recent narrative reviews, the present review restricts its effectiveness synthesis to randomized trials, preserves the paired structure of crossover designs, separates maximum or peak FEV_1_ outcomes from fixed-time-point proxies, pools only the intervention-specific comparisons, and applies the Grading of Recommendations Assessment, Development, and Evaluation (GRADE) to each intervention–comparator–outcome body of evidence. It therefore provides quantitative estimates while defining more narrowly what the evidence can support clinically.

Therefore, this study systematically evaluates the impact of diet and nutritional supplement intervention on EIB, prioritizing the decrease in maximum/peak FEV_1_ within 30 min after exercise or EVH as the primary outcome and labeling fixed-time-point results separately as proxy values. The study synthesized the evidence based on specific interventions, comparisons, outcomes, and clinical backgrounds without calculating the overall effect across nutritional interventions. At the same time, Risk of Bias 2 (RoB 2) and GRADE were used to evaluate the credibility of the evidence, and the athlete’s identity was described according to McKay tier (Sterne et al., 2019; McKay et al., 2022; GRADE Working Group, 2024).

## 2 Methods

### 2.1 Protocol and eligibility criteria

This systematic review was conducted and reported in accordance with the Preferred Reporting Items for Systematic Reviews and Meta-analyses (PRISMA) 2020, with search reporting guided by PRISMA-S (Page et al., 2021a; Page et al., 2021b; Rethlefsen et al., 2021). The protocol was registered in PROSPERO (CRD420261485221; 22 August 2026). Because registration occurred after the final database search and no verifiable public timestamp was available to establish that the protocol or statistical analysis plan preceded the screening, data extraction, or knowledge of the results, the registration was treated as retrospective. The principal analysis decisions and protocol deviations are provided in **Supplementary Table S1**.

The available records confirmed that the database search had been completed before PROSPERO registration. However, a verifiable public timestamp was not available for each detailed analytical decision relative to knowledge of the study results. Accordingly, decisions without a verifiable public timestamp were not described as prospectively preregistered. **Supplementary Table S1** distinguishes the original eligibility and search framework from the analytical decisions finalized during the review, including paired-variance assumptions, proxy outcome labeling, the model used when *k* = 2, and the conditional lycopene analysis.

Randomized parallel-group, crossover, and multi-arm trials were included, whereas non-randomized studies, single-group before-and-after studies, case series, and animal or *in vitro* studies were excluded. Children and adults were eligible when EIB was evaluated by an exercise challenge, EVH, or another accepted indirect bronchial provocation test. Studies with objectively confirmed EIB were prioritized for the main analysis. Studies with incomplete diagnostic details were restricted to narrative or exploratory synthesis and were presented separately, whereas studies based solely on symptoms were excluded. Healthy controls were not included in the quantitative pooling of EIB target effects (Parsons et al., 2013; Hostrup et al., 2024).

Oral dietary modification, electrolyte regulation, nutritional supplements, and functional foods were included. Inhaled or intravenous interventions, pharmacological treatment alone, warm-up, breathing training, environmental equipment interventions, and compound preparations of unknown composition were excluded. Combination preparations were evaluated for additive effects only when randomized comparisons of the additional ingredient could be extracted independently. Athlete status was classified according to the McKay framework, with tiers 2–5 categorized as athletes and samples with insufficient information categorized as mixed or unclear (McKay et al., 2022).

For clarity, eligibility was summarized using PICOS (population, intervention, comparator, outcomes). The population included children or adults evaluated for EIB by exercise challenge, EVH, or another accepted indirect bronchial provocation test, with or without established asthma. Symptom-only diagnosis was excluded. Interventions included oral dietary modification, electrolyte regulation, a nutritional supplement, or a functional food of known composition. Comparators were placebo, a randomized dietary comparator, usual diet when randomized, or another eligible active nutritional condition. Outcomes included maximum or peak percentage fall in FEV_1_ within 30 min, with fixed-time-point values identified as proxies, plus symptoms, rescue medication, exercise performance, inflammatory markers, adherence, and adverse events. Study design included randomized parallel-group, crossover, or multi-arm trials.

### 2.2 Search strategy, study selection, and data extraction

PubMed, Web of Science, Scopus, and CENTRAL (via the Cochrane Library) were searched through to 1 August 2026. The complete search strategies, platforms, search dates, and number of records retrieved are provided in **Supplementary Material S2**. No restrictions were applied by language or year of publication, and gray literature, theses, preprint servers, and trial registries were not searched. The reference lists of the included studies and relevant reviews were also screened. Title/abstract screening and full-text eligibility assessment were performed according to prespecified criteria. Two reviewers (MS and RS) independently screened the titles and abstracts and assessed the full texts. Disagreements were resolved by discussion, with unresolved cases adjudicated by a third reviewer (CS).

PubMed was selected for biomedical coverage, CENTRAL for randomized trial records, and Web of Science and Scopus for multidisciplinary and sports science coverage and citation indexing. Embase, SPORTDiscus, CINAHL, trial registries, dissertations, preprint servers, and other gray literature sources were not searched because an auditable search of these sources was not available for the review period. Their omission may have resulted in missed published or unpublished studies and is considered a limitation.

A structured form was used for data extraction, including the study and report characteristics, design, sample, EIB diagnosis, athlete tier, intervention and comparator, randomization and blinding, crossover sequence and washout period, outcome definition, mean, standard deviation (SD), adherence, adverse events, and funding sources. Directly reported paired effects were prioritized for continuous outcomes. When required, effect estimates were derived from standard errors, 95% confidence intervals (CIs), paired *t* values, *p*-values, or graphical data that could be reliably digitized. When key data were missing, author contact was prioritized, and when the data remained unavailable, only verifiable derivation or narrative synthesis was undertaken (Deeks et al., 2024; Higgins et al., 2024b). Data were extracted independently by MS and RS, with disagreements resolved by discussion and, when required, consultation with CS.

### 2.3 Outcomes, risk of bias, and certainty of evidence

The primary pulmonary function outcome was defined as the maximum or peak percentage fall in FEV_1_ within 0–30 min after exercise or EVH, either reported directly or derived from multiple post-challenge time points. Values reported only at a single fixed time point were retained as proxy outcomes and were clearly labeled. Only one primary time point from each study contributed to a given meta-analysis. Secondary outcomes included area under the FEV_1_ curve, FEV_1_ at individual time points, EIB incidence/protection rate, symptoms, rescue medication, exercise performance, inflammatory markers, adverse events, and adherence. Among the included studies, 14 used an exercise challenge and six used EVH or dry gas hyperventilation. Where reported, the diagnostic thresholds ranged from a fall in FEV_1_ of at least 10% to greater than 15%. Older reports often used the historical term exercise-induced asthma and did not consistently distinguish established asthma from isolated EIB.


$$\text{Maximum FE}{\text{V}}_{1}\text{ decrease }\left(\%\right)\;=\;\left[\left(\text{FE}{\text{V}}_{1}\text{ before exercise }-\text{ lowest FE}{\text{V}}_{1}\text{ after stimulation}\right)/\text{FE}{\text{V}}_{1}\text{ before exercise}\right]\times 100$$

RoB 2 for individually randomized parallel-group trials was applied to parallel trials, and the RoB 2 for crossover trial version was applied to crossover trials, including domains related to period and carryover effects. Certainty of evidence was assessed with GRADE across risk of bias, inconsistency, indirectness, imprecision, and publication/reporting bias. A threshold of 5 percentage points was used only as an operational criterion for judging imprecision and was not treated as a validated minimal clinically important difference. When fewer than 10 studies were available and no direct evidence of missing or selective reporting was identified, publication/reporting bias was recorded as “not assessed.” The RoB 2 assessments and GRADE ratings were completed independently by MS and RS, and disagreements were resolved by consensus, with CS consulted when required.

### 2.4 Data synthesis, heterogeneity, and sensitivity analysis

For crossover trials, directly reported paired effects or participant-level paired data were prioritized. When neither were available, paired variance was reconstructed from the mean and SD of each treatment period. A within-person correlation of *r* = 0.50 was used in the main analysis, with *r* = 0.25 and *r* = 0.75 examined in the sensitivity analyses. Observations from the same participants in different periods were not treated as independent parallel groups. The original paired effect was used for Mickleborough et al. (2013), whereas exact paired variances were calculated from participant-level data for Williams et al. (2017) and Neuman et al. (1999). These estimates therefore were not dependent on the assumed *r* value (Elbourne et al., 2002; Higgins et al., 2024a).

The primary effect measure was the mean difference (MD) with 95%CI, calculated as intervention minus control. Negative values indicated a smaller post-challenge fall in FEV_1_ with the nutritional intervention. Intervention-specific meta-analyses were performed only when the intervention, comparator, outcome, dose/duration, and clinical context were sufficiently comparable and at least two independent trials were available. Random-effects models with restricted maximum likelihood estimation were used for *τ*^2^. Hartung–Knapp adjustment was applied when *k* ≥ 3; when *k* = 2, conventional normal approximation was used, with a fixed-effect model examined in the sensitivity analysis. Caffeine was analyzed separately in the 5- to 6-mg/kg and the 9- to 10-mg/kg dose bands because these two dose bands shared participants and placebo conditions and therefore did not constitute independent bodies of evidence.

Heterogeneity was evaluated using the *Q* test, *I*^2^, *τ*^2^, effect estimates, and clinical differences across studies. Quantitative subgroup analysis was restricted to situations in which each subgroup contained at least three independent and comparable studies. Funnel plots and Egger tests were not performed when fewer than 10 studies were available, and meta-regression was not performed with only two or three studies. Sensitivity analyses addressed objective diagnostic confirmation, exclusion of studies at high risk of bias, model choice, assumed *r* values, challenge method, and data conversion. Only those analyses that were feasible with the available data and remained clinically interpretable were reported (Deeks et al., 2024; Higgins et al., 2024b).

## 3 Results

### 3.1 Study selection

A total of 309 database records were searched (55 in PubMed, 111 in Web of Science, 139 in Scopus, and 4 in CENTRAL). After deleting 46 duplicate records, 263 titles/abstracts were screened, and 49 full texts were obtained. A total of 27 full texts were excluded, and 20 studies (22 reports) were finally included. The main reasons for exclusion were reviews, protocols or conference abstracts (seven articles), ineligible intervention (six articles), non-randomization or unqualified comparison group (six articles), incompatible population or non-EIB specificity (four articles), and unqualified outcomes or no available data (four articles). The 2015 study by Price et al. was excluded because it had a fixed sequence and was not randomly assigned (Price et al., 2015). The study selection process is shown in **Figure 1**.

**Figure 1:**
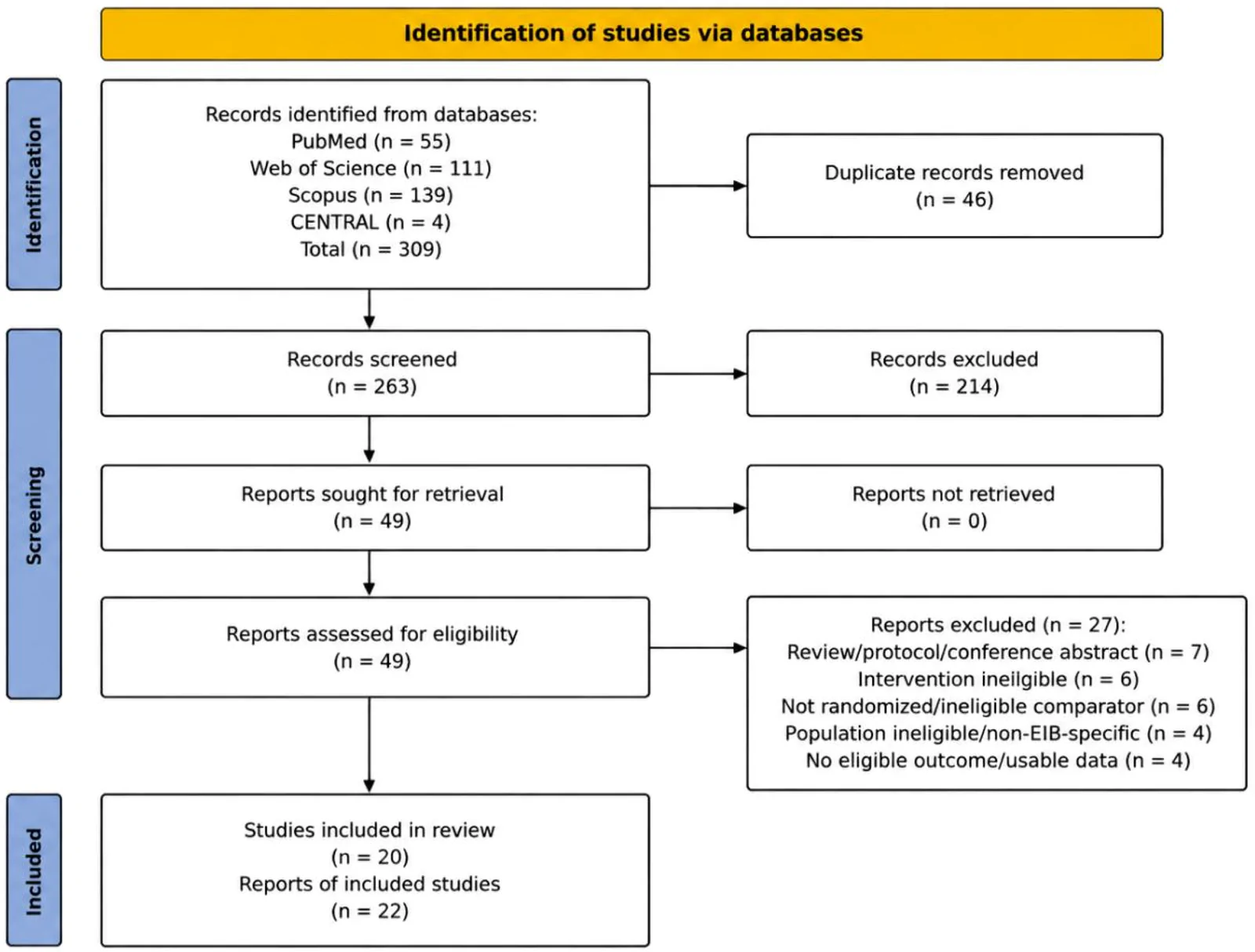
(PRISMA) 2020 study selection flow diagram. Four database sources are shown. Trial registries and gray literature were not searched, and records found through citation searching were not counted separately.

### 3.2 Characteristics of the included evidence and outcome availability

The included studies comprised interventions such as low-sodium/chloride diet, fatty acids, PCSO-524, antioxidant nutrients, caffeine, prebiotics, whey protein, and tangeretin plus whey protein. **Table 1** lists the research-level core characteristics, while **Supplementary Table S4** provides the report-level mapping and analysis positioning. Quantitative or exploratory effect sizes for the main lung function outcomes came from 14 reports. The remaining evidence was entered into single studies or narrative synthesis due to unsatisfactory comparison methods, outcome formats, or pairwise dispersion. There were 11 studies that explicitly described participants as having established asthma. Three athlete cohorts objectively confirmed EIB without clearly establishing underlying asthma, and six studies reported EIB, historical exercise-induced asthma, or exercise-related symptoms while describing asthma status incompletely. Five studies enrolled athlete or physically active cohorts, three of which did not clearly define underlying asthma. **Table 1** summarizes the age and sex when available, asthma and athlete status, EIB ascertainment, intervention dose and duration, background respiratory treatment, and role in the synthesis.

**Table 1:** Clinically oriented characteristics and role of the included studies in the synthesis.

Study and design	Participants	Asthma and athlete status	EIB ascertainment	Intervention and comparator	Duration, background treatment, and role in synthesis
Gotshall et al. (2000); Mickleborough et al. (2001) Randomized double-blind crossover	EIA: *n* = 8; healthy controls also enrolled	Historical EIA; underlying asthma and training level not clearly reported	Exercise challenge; ≥10% fall in FEV_1_ in Gotshall; fixed 15-min proxy	Low-, normal-, and high-sodium diets; chloride manipulation	2 weeks/condition; background medication NR. Low-sodium synthesis compares low with experimental high sodium; healthy controls excluded
Mickleborough et al. (2005) Randomized double-blind crossover	EIA: *n* = 24	Historical EIA; asthma and athlete status incompletely reported	Exercise challenge; fixed 20-min proxy	Low-sodium *vs*. experimental high-sodium diet	2 weeks/condition; background treatment NR. Low-sodium meta-analysis
Mickleborough et al. (2003)) Randomized double-blind crossover	EIB athletes: *n* = 10; 10 athletes without EIB also enrolled	Elite athletes; underlying asthma not clearly defined	Exercise challenge; objective post-exercise FEV_1_ fall	Fish oil (EPA 3.2 g/day + DHA 2.2 g/day) *vs*. olive oil placebo	3 weeks/condition; background treatment NR. EIB group included in the omega-3 meta-analysis
Williams et al. (2017) Randomized double-blind crossover	8 male adults with asthma and HIB; 8 controls without asthma	Established asthma; non-athlete/control status	EVH; peak FEV_1_ fall	*n* - 3 PUFA 6.2 g/day *vs*. placebo for primary comparison	21 days/condition; background treatment not standardized in the synthesis. Omega-3 meta-analysis
Kumar et al. (2016) Randomized double-blind crossover	9 physically active men	Established asthma; physically active	Exercise challenge; lowest post-exercise FEV_1_	EPA/DHA *vs*. placebo	3 weeks/condition; background treatment NR. Omega-3 meta-analysis; high risk of bias
Dreßler et al. (2020) Randomized double-blind parallel	64 analyzed; age, 10–45 years	Exercise-related symptoms with objectively confirmed EIB; asthma status heterogeneous or incompletely reported	Cold-air exercise challenge; >15% fall in FEV_1_	Mixed *n* - 3/*n* - 6 LCPUFA 1.19 g/day *vs*. placebo	4 weeks; registered trial. Single-study estimate
Mickleborough et al. (2013) Randomized double-blind crossover	20 participants	Established asthma with documented HIB	EVH; maximum FEV_1_ fall	PCSO-524 *vs*. placebo	3 weeks/condition with 2-week washout; usual diet. Single-study estimate
Falk et al. (2005) Randomized double-blind crossover	19 young athletes; strict-EIB subgroup: *n* = 12	Athletes recruited for exertional symptoms; underlying asthma not clearly defined	Exercise challenge; *post-hoc* objective subgroup; fixed-time-point proxy	Lycopene 30 mg/day *vs*. placebo	1 week/condition; background treatment NR. Exploratory conditional analysis
Neuman et al. (1999); Neuman et al. (2000) Randomized double-blind trials	Beta-carotene: *n* = 38; responder analysis: *n* = 20; lycopene: *n* = 20	Historical EIA; asthma and athlete status incompletely reported	Exercise challenge; >15% fall after placebo; 8-min proxy	Beta-carotene 64 mg/day or lycopene 30 mg/day *vs*. placebo	1 week; background treatment NR. Post-randomization responder and exploratory lycopene analyses
Tecklenburg et al. (2007) Randomized double-blind crossover	8 participants	Established asthma with documented EIB	Exercise challenge; maximum FEV_1_ fall	Vitamin C 1,500 mg/day *vs*. placebo	2 weeks/condition; 1-week washout; usual diet. Single-study estimate
Duffy and Phillips (1991)) Randomized double-blind crossover	11 men	Objectively documented EIB; underlying asthma not clearly reported	Dry-gas hyperventilation; minimum FEV_1_ over 0–20 min	Caffeine 5 and 10 mg/kg *vs*. placebo	Acute dosing; background medication NR. Two caffeine dose-band meta-analyses
VanHaitsma et al. (2010) Randomized double-blind double-dummy crossover	10 participants	Asthmatic athletes with EIB	Exercise challenge; maximum FEV_1_ fall	Caffeine 6 and 9 mg/kg *vs*. placebo; separate albuterol conditions	Acute dosing; albuterol contrasts analyzed separately. Two caffeine dose-band meta-analyses
Cao et al. (2024) Randomized double-blind parallel	30 professional athletes; 14 women/16 men	Athletes with EIB; underlying asthma not clearly defined	Exercise challenge; maximum fall over 0–30 min	Tangeretin 200 mg/day + whey protein *vs*. whey protein	4 weeks; background treatment NR. Additive effect estimate
Williams et al. (2016) Randomized double-blind crossover	10 adults with asthma and HIB; 8 controls without asthma	Established asthma	EVH; peak FEV_1_ fall	B-GOS prebiotic 5.5 g/day *vs*. placebo	3 weeks/condition; 2-week washout; background treatment NR. Narrative synthesis
Kurti et al. (2016) Randomized single-blind crossover	EIA: *n* = 8; mean age, 22 years; 5 healthy controls	Clinically diagnosed EIA; athlete status not reported	Exercise challenge; FEV_1_ at 1, 5, 15, and 30 min	Vitamin C 500 mg/day + vitamin E 300 IU/day *vs*. placebo	3 weeks/condition; 3-week washout; background treatment NR. Narrative synthesis
Mickleborough et al. (2006) Randomized double-blind crossover	16 participants	Established asthma with documented EIB	Exercise challenge; peak FEV_1_ presented graphically	Fish oil (EPA 3.2 g/day + DHA 2.0 g/day) *vs*. placebo	3 weeks/condition; usual diet. Narrative synthesis because paired percentage data were unavailable
Tecklenburg-Lund et al. (2010) Randomized double-blind multi-arm	20 participants	Established asthma with documented HIB	EVH; peak FEV_1_ fall	Fish oil *vs*. montelukast, followed by combination treatment	3-week periods; active comparator without concurrent placebo. Narrative synthesis
Cohen et al. (1997) Randomized double-blind crossover	20 patients with asthma; age, 7–28 years	Established asthma with EIA	Exercise challenge; ≥15% fall in FEV_1_	Vitamin C vs. placebo	Acute crossover; respiratory medication withheld before challenge. Narrative synthesis
Baumann et al. (2005) Randomized double-blind parallel	18 EIB-positive participants; mean age, 25.2 years	Underlying asthma and athlete status not clearly reported	EVH; ≥10% fall in FEV_1_	Undenatured whey protein 30 g/day *vs*. casein placebo	4 and 8 weeks; background treatment NR. Single-study/narrative synthesis
Kivity et al. (1990) Randomized double-blind crossover	10 participants	Established asthma with EIB	Exercise challenge; post-exercise FEV_1_	Caffeine 3.5 and 7 mg/kg *vs*. placebo	Acute dosing; dose bands outside prespecified meta-analysis. Narrative synthesis

Reports from the same study family are grouped. NR indicates that the item was not reported or was unavailable in the extraction. Exercise-induced asthma (EIA) is retained only when it was the historical term used in the original report. Complete report-level mapping is provided in **Supplementary Table S4**.

*EIB*, exercise-induced bronchoconstriction; *EVH*, eucapnic voluntary hyperpnea; *FEV_1_*, forced expiratory volume in 1 s; *EPA*, eicosapentaenoic acid; *DHA*, docosahexaenoic acid; *LCPUFA*, long-chain polyunsaturated fatty acid; HIB, hyperpnoea-induced bronchoconstriction.

### 3.3 Risk of bias

RoB 2 evaluation was completed on the relevant results of 22 included reports, of which four were parallel groups and 18 were crossover trials. Among the parallel trials, one article was classified as low risk, while three articles were classified as having some concerns. Among the crossover trials, one article was classified as low risk, 13 articles were classified as having some concerns, and four articles were classified as high risk. High-risk ratings arose primarily from missing outcome data, deviations from the intended interventions, and selective reporting. Crossover trials also assessed the treatment sequence, washout periods, baseline recovery, and cycle/carryover effects. The RoB 2 assessments are summarized in **Figure 2**.

**Figure 2:**
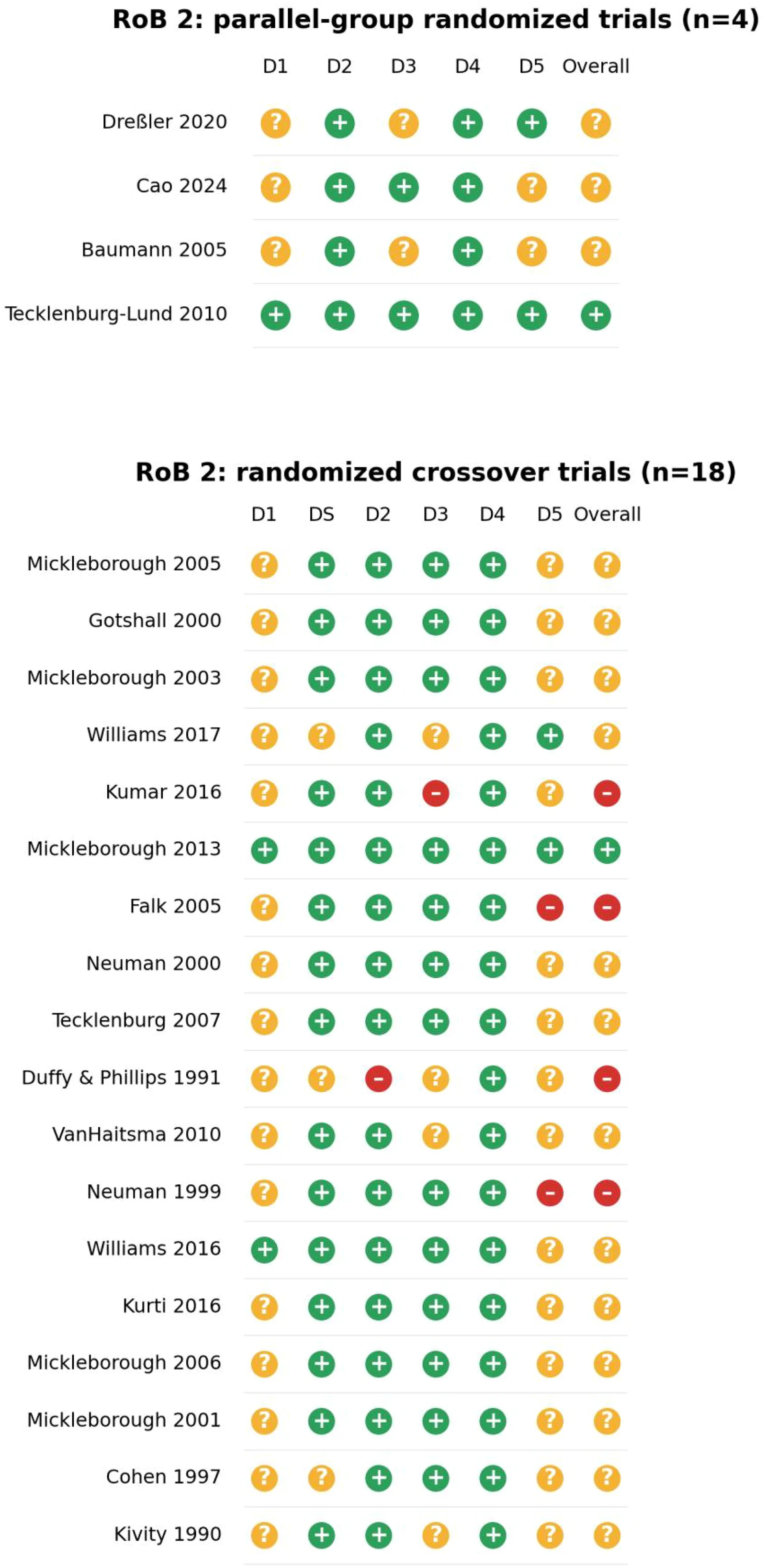
Risk of Bias 2 (RoB 2) assessments for the included randomized trial reports. **(A)** Parallel-group trials (*n* = 4). **(B)** Crossover trials (*n* = 18). *DS* denotes bias arising from period and carryover effects. Domain abbreviations correspond to those in **Supplementary Table S6**.

### 3.4 Main lung function outcomes

All effect sizes are the MD of the intervention condition minus the control condition, and negative values indicate a smaller decrease in FEV_1_ after the intervention. **Table 2** distinguishes between the maximum/peak results and the fixed-time-point surrogate values. Each row represents an independent body of evidence for the “intervention–comparison–primary lung function outcome” and does not calculate the overall effect across nutritional interventions.

**Table 2:** Intervention-specific results of the dietary and nutritional supplement interventions on major lung function outcomes.

Intervention and comparator	Studies (participants)	Outcome, model, and heterogeneity	MD (95%CI), percentage points	GRADE certainty	Interpretation
Low sodium *vs*. experimental high sodium	2 (32)	Fixed-time-point proxy value; random effects	−16.16 (−25.05 to −7.28)	Very low	Risk of bias, outcome indirectness, and imprecision
Standardized omega-3/fish oil *vs*. placebo	3 (27)	Max/peak; REML+HK; *I*^2^ = 85.9%, *τ*^2^ = 8.15	−8.71 (−16.15 to −1.26)	Very low	Risk of bias, inconsistency, and imprecision
Mixed *n* - 3/*n* - 6 LCPUFA *vs*. placebo	1 (64)	0–30 min maximum decrease; single study	−0.60 (−8.89 to 7.69)	Very low	Risk of bias and very serious imprecision
PCSO-524 *vs*. placebo	1 (20)	Maximum decrease; original pairing effect	−14.10 (−21.10 to −7.10)	Moderate	Small sample size per item; downgraded due to imprecision. Manufacturer funding, non-registration, and non-independent replication are reported as background for interpretation, and no automatic inference of publication/reporting bias is used.
Lycopene *vs*. placebo	2 (32)	Fixed-time-point/*post-hoc* subgroup; *I*^2^ = 90.4%, *τ*^2^ = 89.84	−4.95 (−18.76 to 8.86)	Very low	Risk of bias, inconsistency, indirectness, and very serious imprecision
Vitamin C *vs*. placebo	1 (8)	Maximum decline; single study	−6.50 (−10.53 to −2.47)	Very low	Risk of bias and very serious imprecision
Caffeine 5–6 mg/kg *vs*. placebo	2 (21)	0–20 min minimum FEV_1_; *I*^2^ = 0%, *τ*^2^ = 0	−5.59 (−11.13 to −0.04)	Low	Risk of bias and imprecision; sensitive to *r*
Caffeine 9–10 mg/kg *vs*. placebo	2 (21)	0–20 min minimum FEV_1_; *I*^2^ = 0%, *τ*^2^ = 0	−7.96 (−13.25 to −2.68)	Low	Risk of bias and imprecision
Beta-carotene *vs*. placebo (responders)	1 (20 respondents)	8-min surrogate values; exact pairing of participant-level data; selection after randomization	−20.00 (−26.10 to −13.90)	Very low	Post-randomization selection, indirectness, and imprecision
Tangeretin plus whey protein *vs*. whey protein	1 (30)	0–30 min maximum drop; parallel test	−5.34 (−10.70 to 0.02)	Very low	Risk of bias and very serious imprecision (interval includes both important benefit and no effect)

Mean difference (MD) = intervention − control. Negative values indicate that it is beneficial to nutritional intervention. Primary lung function outcomes include the decrease in the maximum/peak forced expiratory volume in 1 s (FEV_1_) from 0 to 30 min and clearly marked fixed-time-point surrogate values. Indirectness due to fixed-time-point proxy values was incorporated into the Grading of Recommendations Assessment, Development, and Evaluation (GRADE) assessment of evidence of low sodium and lycopene.

*LCPUFA*, long-chain polyunsaturated fatty acid; *REML*, restricted maximum likelihood; *HK*, Hartung–Knapp.

Compared with the experimental high-sodium diets, two studies on low-sodium diets showed a reduction in the main FEV_1_ decline outcome (MD = −16.16 percentage points, 95%CI = −25.05 to −7.28), but with both using fixed-time-point proxy values. The comparison group was not a usual dietary intake; thus, the certainty of the evidence was very low. The combined direction of standard *ω* - 3 fatty acids/fish oil was favorable (MD = −8.71, 95%CI = −16.15 to −1.26), but with very high heterogeneity (*I*^2^ = 85.9%). After excluding Kumar et al. (2016), the *I*^2^ dropped to 0%, the combined effect was −10.75 (95%CI = −12.88 to −8.61), and the certainty of the evidence was still very low. A 20-person crossover trial of PCSO-524 showed favorable point estimates; however, independent replication was lacking. The conditional pooling of lycopene contained *post-hoc* strict-EIB subgroups, and the CI crossed the line of no effect.

Vitamin C was only from a single crossover trial of eight people. Caffeine 5–6 mg/kg and 9–10 mg/kg were both potentially beneficial; however, the low-dose results were sensitive to the *r* hypothesis. The two doses were taken from the same group of 21 participants and shared a placebo and cannot be added or considered as independent evidence. The tangeretin study evaluated the additive effect of adding tangeretin on a whey protein background. β-carotene was only analyzed among responders after randomization and cannot represent the overall effect of randomization. The intervention-specific forest plots are shown in **Figure 3**.

**Figure 3:**
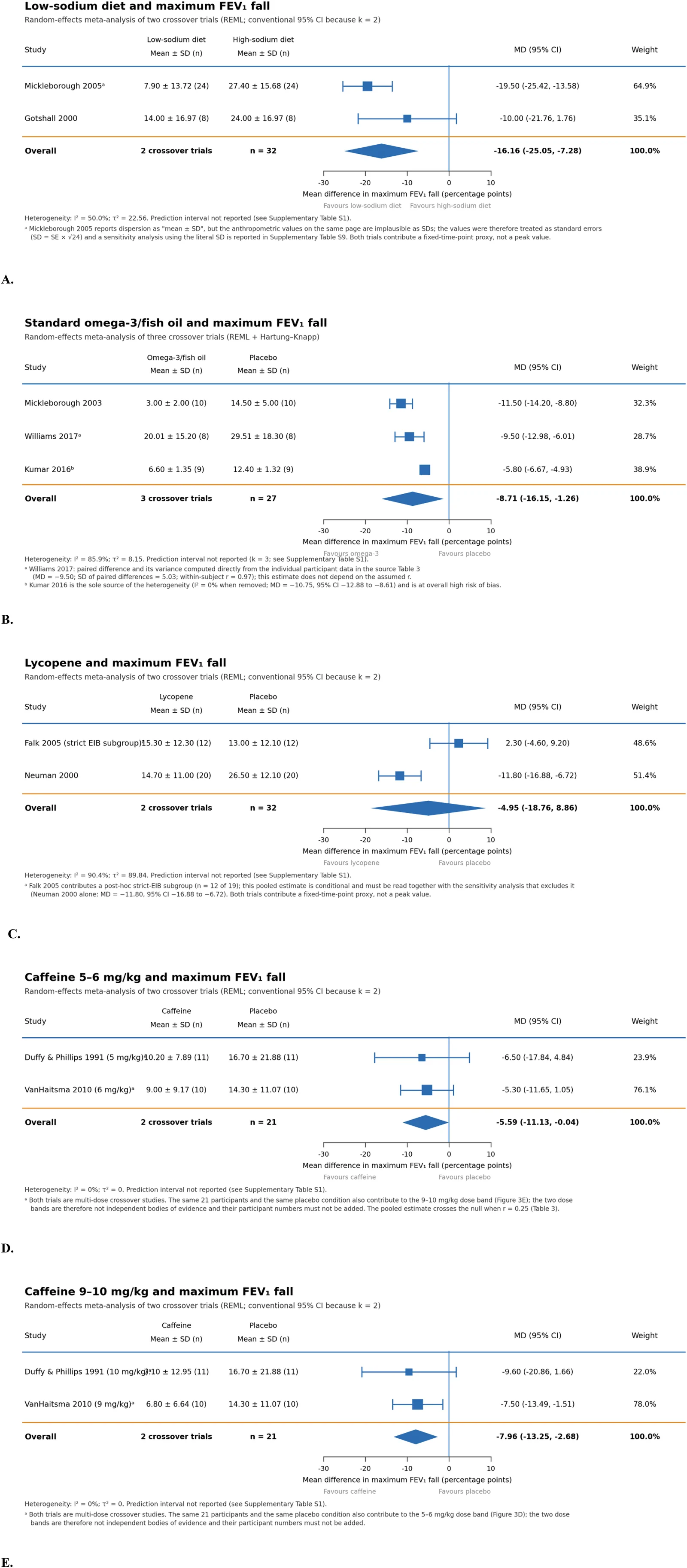
**(A)** Low-sodium diet *versus* experimentally prescribed high-sodium diet: forest plot of the primary pulmonary function outcomes. Negative values favor low sodium. Benefit *versus* usual dietary intake was not tested. **(B)** Standard omega-3 fatty acids/fish oil *versus* placebo: forest plot of primary pulmonary function outcomes. Negative values favor the nutritional intervention. **(C)** Lycopene *versus* placebo: exploratory forest plot including a *post-hoc* strict-exercise-induced bronchoconstriction (EIB) subgroup. **(D)** Caffeine 5–6 mg/kg *versus* placebo: study-by-study forest plot of the primary lung function outcomes. **(E)** Caffeine 9–10 mg/kg *versus* placebo: study-by-study forest plot of the primary lung function outcomes.

### 3.5 Secondary outcomes and safety

The definitions and reporting methods of the symptoms, rescue medications, exercise performance, EIB incidence/protection rates, inflammatory indicators, adverse events, and compliance varied greatly and were not combined across interventions. The majority of trials did not systematically report adverse events, serious adverse events, or withdrawal due to adverse events. Failure to report does not mean that they did not occur; thus, reliable safety conclusions cannot be formed.

Narratively, the selected PCSO-524, vitamin C, and fish oil studies reported improvements in the symptom scores or inflammatory markers. However, the scales, markers, and measurement schedules were inconsistent. Rescue medication use was reported sporadically in the low-sodium, fish oil, and PCSO-524 studies. Exercise performance was examined in only a few trials, and adherence was assessed using differing combinations of capsule counts, diet records, or biomarkers. These outcomes were not pooled. The study-level details are provided in **Supplementary Table S8**.

Particular caution is warranted for caffeine. The EIB trials administered 5–10 mg/kg acutely, equivalent to 350–700 mg for a 70-kg adult. Recent evidence in athletes indicates that acute caffeine intake is associated with dose-related adverse effects, including tachycardia, insomnia, abdominal discomfort, and anxiety, with lower risk at doses of 6 mg/kg or less than at higher doses (Negaresh et al., 2026). Because adverse events and withdrawals were not systematically reported in the EIB trials, the doses examined in these studies should not be interpreted as clinical or athlete-specific recommendations.

### 3.6 Sensitivity analyses for the within-person correlation in crossover trials

Low sodium, standard omega-3/fish oil, and 9–10 mg/kg caffeine had the same direction under different *r* values, with 5–6 mg/kg caffeine having a CI that crossed the line of no effect at *r* = 0.25. The lycopene results were affected by model selection and the inclusion of the strict-EIB subgroup in the meta-set and were therefore only interpreted as exploratory. The core sensitivity analysis is shown in **Table 3**. The sensitivity analyses for the assumed within-person correlation are shown in **Figure 4**.

**Table 3:** Core sensitivity analysis results of the main meta-analysis.

Intervention	Main analysis (*r* = 0.50)	*r* = 0.25	*r* = 0.75	Robustness judgment
Low sodium *vs*. experimental high sodium	−16.16 (−25.05 to −7.28)	−16.88 (−25.20 to −8.55)	−15.45 (−24.66 to −6.25)	Stable direction
Standard omega-3/fish oil	−8.71 (−16.15 to −1.26)	−8.56 (−16.92 to −0.21)	−8.73 (−16.83 to −0.64)	Direction is stable, accuracy is affected by the model
Lycopene	−4.95 (−18.76 to 8.86)	−5.05 (−18.86 to 8.75)	−4.85 (−18.67 to 8.97)	Sensitive to model and meta-subgroups
Caffeine 5–6 mg/kg	−5.59 (−11.13 to −0.04)	−5.63 (−12.22 to 0.97)	−5.51 (−9.66 to −1.36)	Statistical inference is unstable
Caffeine 9–10 mg/kg	−7.96 (−13.25 to −2.68)	−7.96 (−14.19 to −1.74)	−7.96 (−12.10 to −3.83)	Stable direction

The effect size is the median difference (MD) (95%CI), and the unit is percentage points. Negative values indicate that it is beneficial to nutritional intervention. Changing the *r* only affects crossover trials that lack direct pairwise variance and require variance reconstruction based on the correlation coefficients.

**Figure 4:**
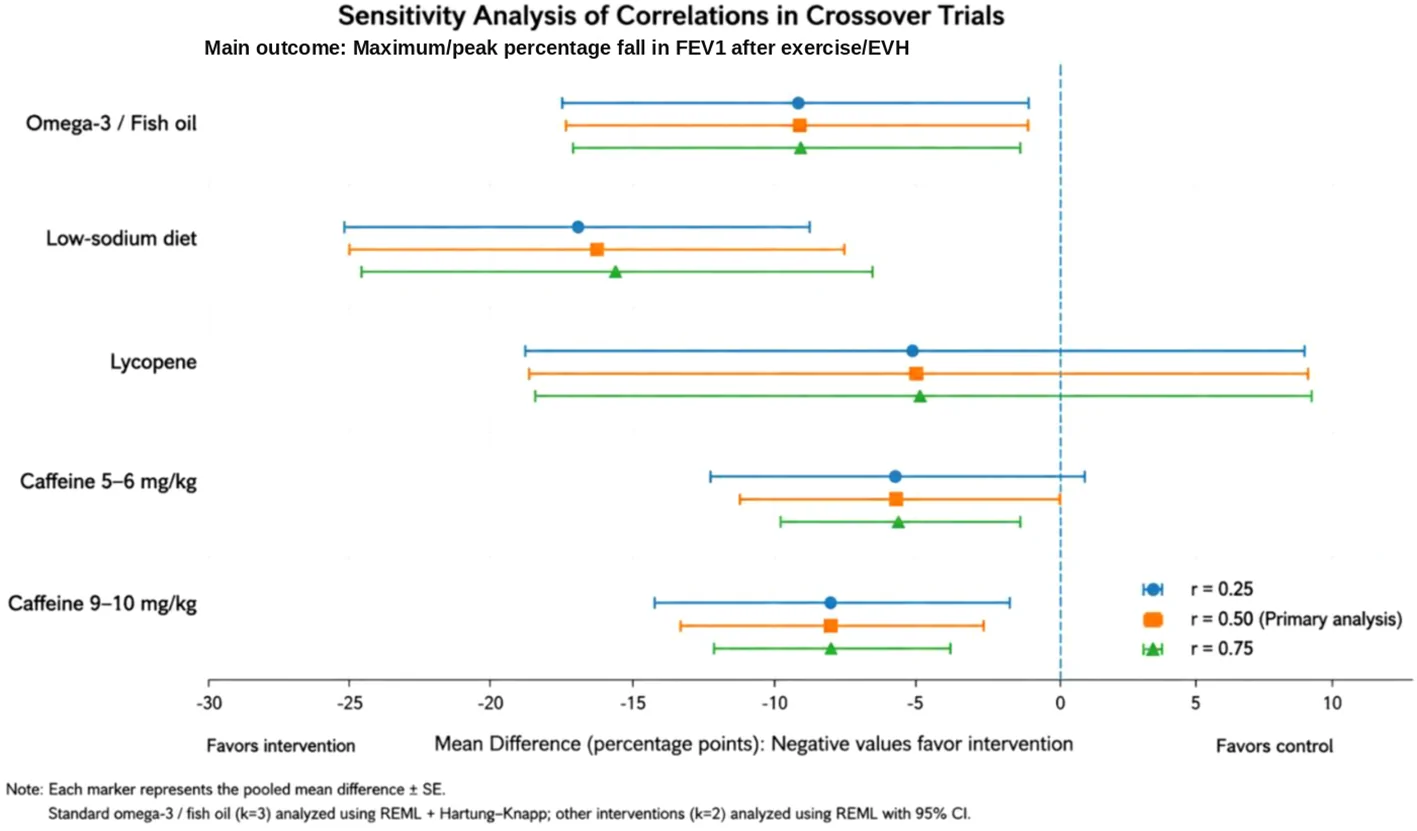
Sensitivity analysis for the assumed within-person correlation in crossover trials. The main analysis used *r* = 0.50, with *r* = 0.25 and *r* = 0.75 as sensitivity settings.

### 3.7 Descriptive stratification by athlete classification

According to the McKay tier framework, four of the independent comparisons in the primary outcomes came from tier 2–5 athletes, four from clear tier 0 to 1/non-athletes, and six from samples with mixed or unclear athlete status. Any specific nutritional intervention did not reach at least three comparable studies at the athlete and non-athlete levels. Therefore, it was only presented descriptively and no stratified meta-analysis or interaction test was performed (McKay et al., 2022; Wilkins et al., 2025). The descriptive stratification by athlete classification is shown in **Figure 5**.

**Figure 5:**
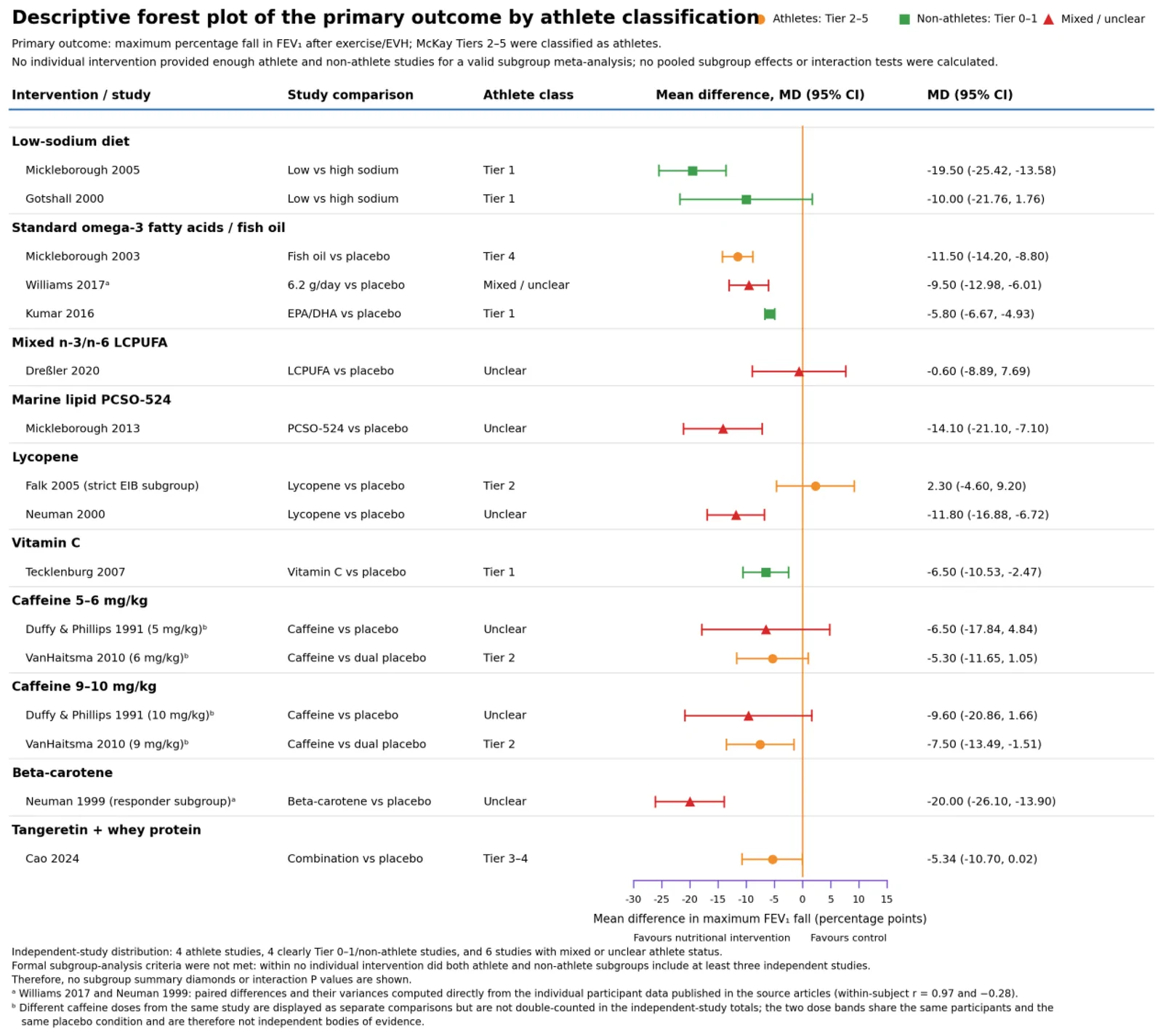
Descriptive forest plot of the primary outcomes with the McKay participant tier. The plot describes the distribution and direction of evidence and is not an inferential subgroup analysis.

### 3.8 Certainty of evidence

GRADE certainty for the primary lung function outcome ranged from very low to moderate. The certainty of evidence for PCSO-524 was rated as moderate; however, this assessment was based on a single 20-participant trial and should not be interpreted as independent replication. The evaluations in each field are shown in **Table 4** and **Supplementary Table S7**.

**Table 4:** Grading of Recommendations Assessment, Development, and Evaluation (GRADE) evidence overview for key lung function outcomes.

Intervention and comparator	Studies (participants)	Certainty	Main limitations	Interpretation
Low sodium *vs*. experimental high sodium	2 (32)	Very low	Proxy value, high sodium comparison, small sample size	The evidence is very uncertain; general recommendations cannot be based on this.
Standardized omega-3/fish oil *vs*. placebo	3 (27)	Very low	Risk of bias, heterogeneity, imprecision	The evidence is very uncertain; general recommendations cannot be based on this.
Mixed *n* - 3/*n* - 6 LCPUFA *vs*. placebo	1 (64)	Very low	Risk of bias, very serious imprecision	The evidence is very uncertain.
PCSO-524 *vs*. placebo	1 (20)	Moderate	Single small sample size; lack of independent replication	One trial suggested a benefit, but still needs to be independently verified.
Lycopene *vs*. placebo	2 (32)	Very low	Meta-subgroups, heterogeneity, proxy values	The evidence is very uncertain.
Vitamin C *vs*. placebo	1 (8)	Very low	Risk of bias, extremely small sample	The evidence is very uncertain.
Caffeine 5–6 mg/kg *vs*. placebo	2 (21)	Low	Risk of bias, imprecision, sensitivity	May be beneficial; the true effects may still differ.
Caffeine 9–10 mg/kg *vs*. placebo	2 (21)	Low	Risk of bias, imprecision	May be beneficial; the true effects may still differ.
Beta-carotene *vs*. placebo (responders)	1 (20)	Very low	Post-randomization selection, proxy values, small samples	Does not represent the overall effect of randomization
Tangeretin plus whey protein *vs*. whey protein	1 (30)	Very low	Risk of bias, CI crosses the line of no effect	Reflects only the additive effect of tangeretin

GRADE evaluates the body of evidence for each specific “intervention–comparison–outcome.” The inconsistency of individual studies cannot be assessed, and the small number of studies also makes it impossible to reliably judge publication bias. Fixed-time-point proxy values are not exactly equivalent to peak outcomes.

*LCPUFA*, long-chain polyunsaturated fatty acid; *CI*, confidence interval.

## 4 Discussion

### 4.1 Main findings

This systematic review included 20 studies (22 reports) covering various types of dietary and nutritional supplement interventions. Low-sodium diets compared with experimentally prescribed high-sodium diets, standard *ω* - 3 fatty acids/fish oil, and specific caffeine doses all showed potentially favorable signals. However, the low-sodium results were proxy values at fixed time points, standard *ω* - 3/fish oil had high heterogeneity, and low-dose caffeine was sensitive to the *r* setting. The favorable signal for PCSO-524 came from only one 20-person trial. The conclusions for lycopene, beta-carotene, and tangeretin plus whey protein were even more uncertain.

The above results cannot be interpreted as “nutritional intervention is overall effective.” The components, doses, durations, comparison groups, challenge regimens, and subject backgrounds of the different interventions differed significantly and were therefore only combined within specific and comparable interventions. Except for PCSO-524, the certainty of the evidence was low or very low. Although PCSO-524 was rated as moderately certain, it also lacked independent replication. Any point estimate should not be directly translated into routine supplementation recommendations (Deeks et al., 2024; GRADE Working Group, 2024; Higgins et al., 2024b). In particular, the moderate certainty rating for PCSO-524 was based on one 20-participant trial without independent replication and should not be interpreted as strong clinical evidence.

### 4.2 Possible biological explanations and clinical significance

Exercise or hyperventilation can induce bronchoconstriction through airway water and heat loss, increased osmotic load, and release of inflammatory mediators (Kippelen et al., 2018; Anderson and Kippelen, 2023). Biological plausibility has been proposed for low-sodium/low-chloride diets, lipid interventions, antioxidant-related nutrients, and caffeine. However, mechanistic plausibility does not substitute for clinical outcome evidence and does not resolve uncertainty arising from heterogeneity, risk of bias, or imprecision (Mokra et al., 2023; Patchen et al., 2023; Song et al., 2023; Zajac and Wojciechowski, 2023; Reddy et al., 2024; Sogard et al., 2025).

Omega-3 fatty acids, PCSO-524, and other lipid interventions may influence inflammatory mediator production or oxidative stress (Patchen et al., 2023; Sogard et al., 2025), whereas antioxidant interventions have been investigated on the premise that oxidative stress contributes to exercise-induced airway responses (Mokra et al., 2023; Zajac and Wojciechowski, 2023). Caffeine may alter short-term respiratory responses through adenosine receptor antagonism and related pharmacological effects (Song et al., 2023; Reddy et al., 2024). However, mechanistic inference cannot substitute for evidence from clinical outcomes. In addition, *post-hoc* strict-EIB subgrouping for lycopene and the post-randomization responder selection for β-carotene introduced analytical uncertainty that could not be resolved by biological plausibility.

The observed pulmonary function estimates were broadly compatible with these hypotheses: favorable omega-3 and PCSO-524 estimates occurred alongside changes in selected inflammatory or oxidative markers in a number of studies, antioxidant studies assessed both FEV_1_ and oxidative or inflammatory outcomes, and caffeine produced short-term FEV_1_ changes consistent with bronchodilation. However, these associations do not demonstrate that the proposed mechanisms caused the pulmonary effects, particularly given the small sample sizes and inconsistent biomarker reporting.

From a clinical perspective, the existing evidence is insufficient to support any dietary or nutritional supplement intervention to replace the standardized evaluation and standard management of EIB (Parsons et al., 2013; Hostrup et al., 2024; Global Initiative for Asthma, 2026). If nutritional strategies are considered, they should be positioned as adjunctive measures after individualized assessment and focus on safety, compliance, background diet, exercise performance, and potential interactions with conventional treatments.

This review uses objective changes in lung function as the primary outcome, distinguishes maximal/peak outcomes from fixed-time-point surrogate values, synthesizes evidence by specific interventions rather than “overall nutritional effects,” and retains the paired structure of crossover trials. The results are generally consistent with the direction of previous narrative evidence, but do not provide reliable evidence to support intervention sequencing or routine recommendations (Elbourne et al., 2002; Deeks et al., 2024; GRADE Working Group, 2024; Higgins et al., 2024b; Higgins et al., 2024a; Sogard et al., 2025).

### 4.3 Heterogeneity and interpretability of results

The differences in the results may have arisen from the elicitation methods, participant characteristics, and intervention exposure. The stimulation properties of the exercise challenge and EVH differed, and the measurement time point, EIB threshold, basic asthma status, training level, background diet and basic treatment were all inconsistent (Parsons et al., 2013; Kippelen et al., 2018). Crossover trials generally lack paired variance, period effects, and carry effect information, and *r* sensitivity analysis can only quantify part of the uncertainty and cannot replace the original paired data. Differences in the established asthma status, athlete training level, age and sex, baseline diet, respiratory medication, intervention formulation and dose, challenge method, diagnostic threshold, and post-challenge measurement schedule may have all modified the observed response and limited the generalizability.

Crossover trials formed an important part of the evidence for this study. The main analysis retained the within-subject pairing structure, and *r* = 0.50 was used for reconstruction when pairwise variance was missing. Robustness was tested with *r* = 0.25 and 0.75 (Elbourne et al., 2002; Higgins et al., 2024a). The inference of low-dose caffeine varies with the setting of *r*, indicating that missing pairing information affects the accuracy of evidence from small samples. PCSO-524 directly uses the original pairing effect. Williams et al. (2017) and Neuman et al. (1999) calculated the pairing variance based on participant-level data and did not rely on the *r* assumption. The unusually small dispersion of Kumar et al. (2016), the dispersion interpretation of Mickleborough et al. (2005), and the CI doubts of Tecklenburg et al. (2007) are all marked in **Supplementary Table S5**. These uncertainties must be interpreted carefully in conjunction with the original report and complete conversion records.

### 4.4 Strengths of this review

Several methodological strengths were incorporated into this review. Objective changes in lung function, rather than symptoms alone, were used as the primary outcome, maximum/peak outcomes were distinguished from fixed-time-point proxies, evidence was synthesized by specific intervention, and crossover trials were analyzed using paired designs. Healthy controls were excluded from the pooled EIB target effects, and athlete status was stratified descriptively rather than subjected to underpowered subgroup inference.

Crossover trials were treated as paired designs, and sensitivity analyses were conducted across alternative within-person correlation assumptions. Fixed regular-diet periods were not treated as randomized control conditions, and healthy controls were not included in the EIB target effect pooling. Athlete status was descriptively stratified according to the McKay tier framework. Because evidence within individual interventions was insufficient, subgroup meta-analysis and interaction testing were not undertaken (McKay et al., 2022; Wilkins et al., 2025).

### 4.5 Limitations

The principal limitation was the small evidence base. Majority of the meta-analyses included only two or three studies, which precluded reliable assessment of publication bias, meta-regression, and dose–response relationships. Embase, SPORTDiscus, CINAHL, gray literature, and trial registries were not searched, and records identified through citation tracing were not counted separately. The protocol was registered retrospectively in PROSPERO (CRD420261485221; 22 August 2026), after completion of the final database search, and thus the limitations associated with the absence of prospective registration remain. In addition, fixed-time-point proxy outcomes coexisted with maximum/peak outcomes, reducing comparability across studies.

RoB 2 assessment was completed for all 22 included reports. However, only 14 reports contributed quantitative or exploratory effect estimates for the primary outcome. Some key data required variance reconstruction or conversion, and uncertainties in the original reports by Mickleborough et al. (2005), Tecklenburg et al. (2007), and Kumar et al. (2016) could not be fully resolved. These procedures were documented in the **Supplementary Material**, but could not replace verification against original participant-level data. The report-level full-text screening records, extraction tables, and analysis code are provided as **Supplementary Materials** to support transparency and reproducibility.

### 4.6 Implications for clinical practice and future research

Existing evidence is more appropriate for setting future research priorities than supporting the routine use of a nutritional supplement to prevent EIB. Low-sodium strategies, standard omega-3 fatty acids/fish oil, PCSO-524, and specific caffeine doses are only candidate interventions for further validation and do not constitute clinical use or dosage recommendations (Hostrup et al., 2024; Sogard et al., 2025).

Low sodium compared with an appropriate usual-intake comparator, standard omega-3/fish oil, PCSO-524, and selected caffeine doses may warrant confirmatory trials. Evidence for vitamin C, lycopene, beta-carotene, mixed fatty acid products, prebiotics, whey protein, and tangeretin remains extremely limited, indirect, or inconsistent for practical recommendations. PCSO-524 requires independent replication, and caffeine requires systematic safety assessment before either could be considered clinically.

Future trials should be conducted in populations with objectively confirmed EIB and should use preregistered protocols with clearly described randomization, blinding, allocation concealment, washout periods, and statistical analysis plans. The challenge conditions, FEV_1_ measurement timings, primary outcome calculations, paired variance, background treatments and diet, training levels, adherence, adverse events, and exercise performance should be reported in sufficient detail to support replication and evidence synthesis.

## 5 Conclusion

Low-sodium diets compared with experimentally prescribed high-sodium diets, standard omega-3 fatty acids/fish oils, and specific caffeine doses present potentially beneficial signals. However, the evidence comes primarily from small studies and is limited by proxy outcomes, heterogeneity, risk of bias, or missing paired data. Although PCSO-524 was rated as moderately certain, it was still based on only one 20-person trial. At this stage, no nutritional intervention can be recommended as a routine preventive measure or as an alternative to standard treatment for EIB, and there is insufficient safety evidence.

## Data Availability

The original contributions presented in the study are included in the article/**Supplementary Material**. Further inquiries can be directed to the corresponding author.
