# Supplementary material for: Dietary modification and nutritional supplements for exercise-induced bronchoconstriction: a systematic review and meta-analysis
Source: Front Physiol. 2026 Oct 13;17:1975449. doi: 10.3389/fphys.2026.1975449 (PMC13710025; doi:10.3389/fphys.2026.1975449)
Supplement: Supplementary file 1 [file SupplementaryFile1.docx]

# S1. Protocol status, registration timing, and analytical deviations

Table S1. Protocol status, registration timing, and analytical deviations.

| **Decision area** | **Timing/status** | **Details** |
| --- | --- | --- |
| Eligibility and search framework | Documented without a public timestamp | Defined eligible randomized designs, oral nutritional interventions, objective EIB testing, the principal pulmonary outcome, and main exclusions; its timing relative to knowledge of individual study results cannot be independently verified. |
| PROSPERO registration | Retrospective | Registered as CRD420261485221 on August 22, 2026, after the final database search on August 1, 2026; it is not presented as prospective registration. |
| Crossover paired analysis | Finalized during analysis | Direct paired effects or participant-level data were preferred; when paired variance was unavailable, r=0.50 was used, with r=0.25 and r=0.75 in sensitivity analyses. |
| Conditional and exploratory analyses | Post hoc | The Falk 2005 strict-EIB subgroup was pooled conditionally; the Neuman 1999 responder analysis does not represent the full randomized population. |
| Model for k=2 meta-analyses | Analytical deviation | A conventional normal approximation was used because Hartung-Knapp inference was unstable with two studies; fixed-effect sensitivity analyses were also examined. |
| GRADE operational rules | Defined during analysis | Operational rules for imprecision and publication/reporting bias are reported in the Methods and Table S7 and are not described as prospectively preregistered. |

Note: The review was registered in PROSPERO (CRD420261485221; August 22, 2026) after the final database search. The available records do not provide a public timestamp that establishes when each detailed analytical decision was finalized relative to knowledge of the study results. The table therefore distinguishes the initial eligibility and search framework from decisions finalized during analysis.

# S2. Information sources, search records, and complete search strategies

Table S2. Information sources and search records.

| **Database/source** | **Platform** | **Coverage** | **Records retrieved** | **Export file/format** | **Rationale or limitation** |
| --- | --- | --- | --- | --- | --- |
| PubMed/MEDLINE | PubMed | From inception to August 1, 2026 | 55 | pm.txt | Biomedical coverage; no language or year restriction |
| Web of Science Core Collection | Web of Science | From inception to August 1, 2026 | 111 | wos.xls | Multidisciplinary and citation-index coverage, including sport science |
| Scopus | Scopus | From inception to August 1, 2026 | 139 | Scopus CSV export | Multidisciplinary title, abstract, and keyword coverage, including sport science |
| Cochrane Central Register of Controlled Trials (CENTRAL) | Cochrane Library | From inception to August 1, 2026 | 4 | citation-export.txt | Randomized and quasi-randomized trial records |
| Backward citation searching | Included studies and relevant reviews | Completed; date not recorded separately | Not counted separately | Project records | Source count was not archived separately |
| Trial registries | ClinicalTrials.gov/WHO ICTRP | Not searched | — | — | May have missed ongoing or unpublished trials |

Note: Table S2 reports the database platform, coverage, search date, records retrieved, and export format. Embase, SPORTDiscus, CINAHL, trial registries, dissertations, preprints, and other gray-literature sources were not searched because an auditable search of these sources was not available for the review period. Their omission may have resulted in missed published or unpublished studies.

## S2.1 PubMed/MEDLINE (55 hits)

**(
 "Asthma, Exercise-Induced"[Mesh]
 OR "exercise-induced bronchoconstriction"[tiab]
 OR "exercise induced bronchoconstriction"[tiab]
 OR "exercise-related bronchoconstriction"[tiab]
 OR "exercise induced bronchospasm"[tiab]
 OR "exercise-induced bronchospasm"[tiab]
 OR "exercise-induced asthma"[tiab]
 OR "exercise induced asthma"[tiab]
 OR "postexercise bronchoconstriction"[tiab]
 OR "post-exercise bronchoconstriction"[tiab]
 OR "hyperpnea-induced bronchoconstriction"[tiab]
 OR "hyperpnoea-induced bronchoconstriction"[tiab]
 OR "hyperventilation-induced bronchoconstriction"[tiab]
)
AND
(
 "Diet Therapy"[Mesh] OR "Dietary Supplements"[Mesh]
 OR "Diet"[Mesh] OR "Food"[Mesh]
 OR "Sodium Chloride, Dietary"[Mesh]
 OR "Fish Oils"[Mesh] OR "Fatty Acids, Omega-3"[Mesh]
 OR "Ascorbic Acid"[Mesh] OR "Vitamin D"[Mesh]
 OR "Vitamin E"[Mesh] OR "Caffeine"[Mesh]
 OR "Antioxidants"[Mesh] OR "Carotenoids"[Mesh]
 OR "Whey Proteins"[Mesh]
 OR diet*[tiab] OR nutrition*[tiab] OR nutraceutical*[tiab]
 OR supplement*[tiab] OR "dietary intervention"[tiab]
 OR "dietary modification"[tiab] OR "low sodium diet"[tiab]
 OR "low-sodium diet"[tiab] OR "low salt diet"[tiab]
 OR "low-salt diet"[tiab] OR "sodium restriction"[tiab]
 OR "salt restriction"[tiab] OR "dietary sodium"[tiab]
 OR "dietary salt"[tiab] OR "dietary chloride"[tiab]
 OR "fish oil"[tiab] OR "fish oils"[tiab]
 OR omega-3[tiab] OR "omega 3"[tiab]
 OR "n-3 fatty acid"[tiab] OR "n-3 fatty acids"[tiab]
 OR PUFA*[tiab] OR eicosapentaenoic[tiab]
 OR docosahexaenoic[tiab] OR EPA[tiab] OR DHA[tiab]
 OR "ascorbic acid"[tiab] OR "vitamin C"[tiab]
 OR "vitamin D"[tiab] OR cholecalciferol[tiab]
 OR "vitamin E"[tiab] OR tocopherol*[tiab]
 OR caffeine[tiab] OR coffee[tiab] OR antioxidant*[tiab]
 OR lycopene[tiab] OR carotenoid*[tiab]
 OR "beta-carotene"[tiab] OR "beta carotene"[tiab]
 OR flavonoid*[tiab] OR tangeretin[tiab]
 OR "whey protein"[tiab] OR "whey proteins"[tiab]
 OR "undenatured whey"[tiab] OR "cysteine donor"[tiab]
 OR Immunocal[tiab] OR "marine lipid"[tiab]
 OR "marine lipid fraction"[tiab]
 OR "green-lipped mussel"[tiab] OR "green lipped mussel"[tiab]
 OR PCSO-524[tiab] OR Smartfish[tiab] OR NutriFriend[tiab]
)
AND
(
 randomized controlled trial[pt] OR controlled clinical trial[pt]
 OR clinical trial[pt] OR random*[tiab] OR placebo*[tiab]
 OR trial*[tiab] OR crossover[tiab] OR "crossover"[tiab]
 OR "cross over"[tiab] OR parallel[tiab] OR blind*[tiab]
 OR controlled[tiab]
)
NOT (animals[mh] NOT humans[mh])**

## S2.2 Cochrane CENTRAL (4 hits)

**#1 [mh "Asthma, Exercise-Induced"]
#2 (((exercise-induced OR "exercise induced" OR exercise-related
 OR "exercise related" OR postexercise OR "post exercise")
 NEAR/3 (bronchoconstrict* OR bronchospasm* OR asthma*))):ti,ab,kw
#3 (((hyperpnea OR hyperpnoea OR hyperventilation OR EVH)
 NEAR/3 bronchoconstrict*)):ti,ab,kw
#4 #1 OR #2 OR #3
#5 [mh "Diet Therapy"] OR [mh "Dietary Supplements"] OR [mh Diet]
 OR [mh Food] OR [mh "Sodium Chloride, Dietary"]
 OR [mh "Fish Oils"] OR [mh "Fatty Acids, Omega-3"]
 OR [mh "Ascorbic Acid"] OR [mh "Vitamin D"]
 OR [mh "Vitamin E"] OR [mh Caffeine]
 OR [mh Antioxidants] OR [mh Carotenoids] OR [mh "Whey Proteins"]
#6 (diet* OR nutrition* OR nutraceutical* OR supplement*
 OR "dietary intervention" OR "dietary modification"
 OR "low sodium diet" OR "low-sodium diet"
 OR "low salt diet" OR "low-salt diet"
 OR "sodium restriction" OR "salt restriction"
 OR "dietary sodium" OR "dietary salt" OR "dietary chloride"
 OR "fish oil" OR "fish oils" OR omega-3 OR "omega 3"
 OR "n-3 fatty acid*" OR PUFA* OR eicosapentaenoic
 OR docosahexaenoic OR EPA OR DHA OR "ascorbic acid"
 OR "vitamin C" OR "vitamin D" OR cholecalciferol
 OR "vitamin E" OR tocopherol* OR caffeine OR coffee
 OR antioxidant* OR lycopene OR carotenoid*
 OR beta-carotene OR "beta carotene" OR flavonoid*
 OR tangeretin OR "whey protein*" OR "undenatured whey"
 OR "cysteine donor*" OR Immunocal OR "marine lipid*"
 OR "green-lipped mussel*" OR "green lipped mussel*"
 OR PCSO-524 OR Smartfish OR NutriFriend):ti,ab,kw
#7 #5 OR #6
#8 #4 AND #7**

## S2.3 Web of Science Core Collection (111 hits)

**TS=(
 ((("exercise-induced" OR "exercise induced" OR "exercise-related"
 OR "exercise related" OR postexercise OR "post exercise") NEAR/3
 (bronchoconstrict* OR bronchospasm* OR asthma*)))
 OR (((hyperpnea OR hyperpnoea OR hyperventilation OR EVH) NEAR/3
 bronchoconstrict*))
)
AND TS=(
 diet* OR nutrition* OR nutraceutical* OR supplement*
 OR "dietary intervention" OR "dietary modification"
 OR "low sodium diet" OR "low-sodium diet"
 OR "low salt diet" OR "low-salt diet"
 OR "sodium restriction" OR "salt restriction"
 OR "dietary sodium" OR "dietary salt" OR "dietary chloride"
 OR "fish oil" OR "fish oils" OR omega-3 OR "omega 3"
 OR "n-3 fatty acid*" OR PUFA* OR eicosapentaenoic
 OR docosahexaenoic OR EPA OR DHA OR "ascorbic acid"
 OR "vitamin C" OR "vitamin D" OR cholecalciferol
 OR "vitamin E" OR tocopherol* OR caffeine OR coffee
 OR antioxidant* OR lycopene OR carotenoid*
 OR beta-carotene OR "beta carotene" OR flavonoid*
 OR tangeretin OR "whey protein*" OR "undenatured whey"
 OR "cysteine donor*" OR Immunocal OR "marine lipid*"
 OR "green-lipped mussel*" OR PCSO-524 OR Smartfish OR NutriFriend
)
AND TS=(
 random* OR placebo* OR trial* OR crossover OR "crossover"
 OR "cross over" OR parallel OR blind* OR controlled
)**

## S2.4 Scopus (139 hits)

**TITLE-ABS-KEY(
 ((("exercise-induced" OR "exercise induced" OR "exercise-related"
 OR "exercise related" OR postexercise OR "post exercise") W/3
 (bronchoconstrict* OR bronchospasm* OR asthma*)))
 OR (((hyperpnea OR hyperpnoea OR hyperventilation OR EVH) W/3
 bronchoconstrict*))
)
AND TITLE-ABS-KEY(
 diet* OR nutrition* OR nutraceutical* OR supplement*
 OR "dietary intervention" OR "dietary modification"
 OR "low sodium diet" OR "low-sodium diet"
 OR "low salt diet" OR "low-salt diet"
 OR "sodium restriction" OR "salt restriction"
 OR "dietary sodium" OR "dietary salt" OR "dietary chloride"
 OR "fish oil" OR "fish oils" OR omega-3 OR "omega 3"
 OR "n-3 fatty acid" OR "n-3 fatty acids" OR PUFA*
 OR eicosapentaenoic OR docosahexaenoic OR EPA OR DHA
 OR "ascorbic acid" OR "vitamin C" OR "vitamin D"
 OR cholecalciferol OR "vitamin E" OR tocopherol*
 OR caffeine OR coffee OR antioxidant* OR lycopene
 OR carotenoid* OR beta-carotene OR "beta carotene"
 OR flavonoid* OR tangeretin OR "whey protein"
 OR "whey proteins" OR "undenatured whey"
 OR "cysteine donor" OR Immunocal OR "marine lipid"
 OR "marine lipid fraction" OR "green-lipped mussel"
 OR "green lipped mussel" OR PCSO-524 OR Smartfish OR NutriFriend
)
AND TITLE-ABS-KEY(
 random* OR placebo* OR trial* OR crossover OR "crossover"
 OR "cross over" OR parallel OR blind* OR controlled
)**

# S3. Reasons for exclusion after full-text assessment

Table S3. Reasons for exclusion after full-text assessment.

| **Reason for exclusion after full-text assessment** | **Reports, n** |
| --- | --- |
| Review, protocol, or conference abstract | 7 |
| Ineligible intervention | 6 |
| Nonrandomized design or ineligible comparator | 6 |
| Ineligible population or not specific to EIB | 4 |
| Ineligible outcome or unavailable data | 4 |
| Total | 27 |

Note: Reasons and totals correspond to the PRISMA flow diagram in the main manuscript. Report-level screening records for all 49 full-text articles should be deposited in the open repository.

Key exclusion: Price et al. (2015) used a fixed intervention sequence without random allocation and was therefore excluded as a nonrandomized study.

# S4. Included study-report mapping and analytical details

Table S4. Included study-report mapping and role in the synthesis.

| **Study ID** | **Report(s) and report ID(s)** | **Role in synthesis** |
| --- | --- | --- |
| ST01 | R01 Gotshall 2000; R02 Mickleborough 2001 | Low-sodium meta-analysis (Gotshall); companion report used for contextual information |
| ST02 | R03 Mickleborough 2005 | Low-sodium meta-analysis |
| ST03 | R04 Mickleborough 2003 | Standard omega-3 meta-analysis |
| ST04 | R05 Williams 2017 | Standard omega-3 meta-analysis |
| ST05 | R06 Kumar 2016 | Standard omega-3 meta-analysis |
| ST06 | R07 Dreßler 2020 | Single-trial estimate |
| ST07 | R08 Mickleborough 2013 | Single-trial estimate |
| ST08 | R09 Falk 2005 | Exploratory conditional analysis using a post hoc strict-EIB subgroup |
| ST09 | R10 Neuman 1999; R11 Neuman 2000 | Exploratory lycopene analysis; exploratory post-randomization beta-carotene responder analysis |
| ST10 | R12 Tecklenburg 2007 | Single-trial estimate |
| ST11 | R13 Duffy and Phillips 1991 | Two caffeine dose-band meta-analyses |
| ST12 | R14 VanHaitsma 2010 | Two caffeine dose-band meta-analyses |
| ST13 | R15 Cao 2024 | Single-trial additive-effect estimate |
| ST14 | R16 Williams 2016 | Narrative synthesis |
| ST15 | R17 Kurti 2016 | Narrative synthesis |
| ST16 | R18 Mickleborough 2006 | Narrative synthesis; percentage FEV₁ fall was presented only graphically, and no figure digitization was undertaken |
| ST17 | R19 Tecklenburg-Lund 2010 | Narrative synthesis; active comparison with montelukast and no concurrent placebo arm |
| ST18 | R20 Cohen 1997 | Narrative synthesis; whole-sample mean and dispersion for percentage FEV₁ fall were unavailable |
| ST19 | R21 Baumann 2005 | Single-trial estimate/narrative synthesis |
| ST20 | R22 Kivity 1990 | Narrative synthesis; absolute FEV₁ reported and doses fell outside the prespecified working dose bands |

Note: Table S4 provides the study-report mapping and the role of each report in the synthesis. Complete report-level extraction fields and source locations should be deposited in the open repository.

# S5. Quantitative synthesis and data processing for primary outcomes

Table S5. Data processing for the primary quantitative outcomes.

| **Study** | **Randomized contrast** | **Primary outcome and variance handling** | **Role in synthesis** |
| --- | --- | --- | --- |
| Mickleborough 2005 | Low sodium vs. high sodium; 2 weeks/condition | Exercise challenge; 20-min value/lowest FEV₁ within 0-30 min; condition-level data. The report labels values as mean ± SD, but the within-report height dispersion (164 ± 1.5 cm; n=24) suggests that some dispersions may be SE. The main analysis treated them as SE (SD=SE×√24); a literal-SD sensitivity analysis should be reported with the open data and code. | Primary low-sodium meta-analysis |
| Gotshall 2000 | Low sodium vs. high sodium; 2 weeks/condition | Exercise challenge; fixed 15-min value; condition-level data; paired variance reconstructed across the prespecified correlations | Primary low-sodium meta-analysis |
| Mickleborough 2003 | Fish oil vs. olive-oil placebo; 3 weeks/condition | Exercise challenge; maximum FEV₁ fall; condition-level data; paired variance reconstructed across the prespecified correlations | Primary omega-3 meta-analysis |
| Williams 2017 | n-3 PUFA 6.2 g/day vs. placebo; 21 days/condition | EVH; peak FEV₁ fall. Participant-level data from Table 3 yielded MD=−9.50, SD of paired differences=5.03, SE=1.78, and within-person r=0.97; no assumed correlation was required. | Primary omega-3 meta-analysis |
| Kumar 2016 | EPA/DHA vs. placebo; 3 weeks/condition | Exercise challenge; lowest postexercise FEV₁; condition-level SEMs of 0.44% and 0.45% (n=9) imply SDs of approximately 1.3 percentage points. Paired variance was reconstructed; leave-one-out results should be reported with the open data and code. | Primary omega-3 meta-analysis; high risk of bias |
| Dreßler 2020 | Mixed n-3/n-6 LCPUFA vs. placebo; 4 weeks | Exercise challenge; maximum FEV₁ fall within 0-30 min; parallel-group endpoint estimate | Single-trial estimate |
| Mickleborough 2013 | PCSO-524 vs. placebo; 3 weeks/condition | EVH; maximum FEV₁ fall; directly reported paired effect and 95% CI | Single-trial estimate |
| Falk 2005 | Lycopene 30 mg/day vs. placebo; 1 week/condition | Exercise challenge; fixed 5-min value in a post hoc strict-EIB subgroup; condition-level data; paired variance reconstructed across the prespecified correlations | Exploratory conditional analysis/protocol deviation |
| Neuman 2000 | Lycopene 30 mg/day vs. placebo; 1 week | Exercise challenge; fixed 8-min value; condition-level data; paired variance reconstructed across the prespecified correlations | Exploratory lycopene analysis |
| Tecklenburg 2007 | Vitamin C 1,500 mg/day vs. placebo; 2 weeks/condition | Exercise challenge; maximum FEV₁ fall. Condition-level 95% CIs were converted to SDs. The placebo interval (−18.6 to −12.3 around a mean of −12.9) is asymmetric and may contain a reporting error; the main analysis used the values as printed. A symmetry-based sensitivity analysis should be reported with the open data and code. | Single-trial estimate |
| Duffy and Phillips 1991 | Caffeine 5 and 10 mg/kg vs. placebo; acute | Dry-gas hyperventilation; minimum FEV₁ within 0-20 min; condition-level data; paired variance reconstructed across the prespecified correlations | Two caffeine dose-band meta-analyses; high risk of bias |
| VanHaitsma 2010 | Caffeine 6 and 9 mg/kg vs. placebo; acute | Exercise challenge; postexercise minimum FEV₁; condition-level data; paired variance reconstructed across the prespecified correlations | Two caffeine dose-band meta-analyses |
| Neuman 1999 | Beta-carotene 64 mg/day vs. placebo; 1 week | Exercise challenge; fixed 8-min value. Participant-level data for 20 responders yielded MD=−20.00, SD of paired differences=13.92, SE=3.11, and within-person r=−0.28; the estimate excludes nonresponders selected after randomization. | Exploratory responder analysis only |
| Cao 2024 | Tangeretin plus whey protein vs. whey-protein control; 4 weeks | Exercise challenge; maximum FEV₁ fall within 3-30 min; parallel-group endpoint estimate. Baseline imbalance (16.12 ± 4.97 vs. 17.60 ± 8.36) should be examined in a change-score sensitivity analysis with the open data and code. | Single-trial additive-effect estimate |

Note: Table S5 summarizes primary-outcome sources and variance handling. Participant-level values, conversion formulas, raw extraction tables, sensitivity analyses, and analysis code should be deposited in the open repository.

# S6. RoB 2 risk-of-bias assessments

Table S6A. RoB 2 domain-level judgments for parallel-group randomized trials.

| **Study report** | **D1** | **D2** | **D3** | **D4** | **D5** | **Overall** |
| --- | --- | --- | --- | --- | --- | --- |
| Dreßler 2020 | Some concerns | Low | Some concerns | Low | Low | Some concerns |
| Cao 2024 | Some concerns | Low | Low | Low | Some concerns | Some concerns |
| Baumann 2005 | Some concerns | Low | Some concerns | Low | Some concerns | Some concerns |
| Tecklenburg-Lund 2010 | Low | Low | Low | Low | Low | Low |

Table S6B. RoB 2 domain-level judgments for randomized crossover trials.

| **Study report** | **D1** | **DS** | **D2** | **D3** | **D4** | **D5** | **Overall** |
| --- | --- | --- | --- | --- | --- | --- | --- |
| Mickleborough 2005 | Some concerns | Low | Low | Low | Low | Some concerns | Some concerns |
| Gotshall 2000 | Some concerns | Low | Low | Low | Low | Some concerns | Some concerns |
| Mickleborough 2003 | Some concerns | Low | Low | Low | Low | Some concerns | Some concerns |
| Williams 2017 | Some concerns | Some concerns | Low | Some concerns | Low | Low | Some concerns |
| Kumar 2016 | Some concerns | Low | Low | High | Low | Some concerns | High |
| Mickleborough 2013 | Low | Low | Low | Low | Low | Low | Low |
| Falk 2005 | Some concerns | Low | Low | Low | Low | High | High |
| Neuman 2000 | Some concerns | Low | Low | Low | Low | Some concerns | Some concerns |
| Tecklenburg 2007 | Some concerns | Low | Low | Low | Low | Some concerns | Some concerns |
| Duffy and Phillips 1991 | Some concerns | Some concerns | High | Some concerns | Low | Some concerns | High |
| VanHaitsma 2010 | Some concerns | Low | Low | Some concerns | Low | Some concerns | Some concerns |
| Neuman 1999 | Some concerns | Low | Low | Low | Low | High | High |
| Williams 2016 | Low | Low | Low | Low | Low | Some concerns | Some concerns |
| Kurti 2016 | Some concerns | Low | Low | Low | Low | Some concerns | Some concerns |
| Mickleborough 2006 | Some concerns | Low | Low | Low | Low | Some concerns | Some concerns |
| Mickleborough 2001 | Some concerns | Low | Low | Low | Low | Some concerns | Some concerns |
| Kivity 1990 | Some concerns | Low | Low | Some concerns | Low | Some concerns | Some concerns |
| Cohen 1997 | Some concerns | Some concerns | Low | Low | Low | Some concerns | Some concerns |

RoB 2 was applied to 22 included reports: four parallel-group trials and 18 crossover trials. Judgments relate to the outcomes used in this review. The crossover version also assesses bias arising from period and carryover effects; Tables S6A and S6B provide report-level domain judgments.

Figure S1A. Distribution of RoB 2 domain judgments for parallel-group randomized trials (n=4). Report-level judgments are shown in Figure 2A of the main manuscript and Table S6A.

Figure S1B. Distribution of RoB 2 domain judgments for randomized crossover trials (n=18). DS denotes bias arising from period and carryover effects; report-level judgments are shown in Figure 2B of the main manuscript and Table S6B.

# S7. GRADE certainty-of-evidence assessments

Table S7. GRADE judgments for the primary pulmonary-function outcomes.

| **Body of evidence** | **Risk of bias** | **Inconsistency** | **Indirectness** | **Imprecision** | **Publication/reporting bias** | **Certainty** |
| --- | --- | --- | --- | --- | --- | --- |
| Low sodium vs. experimental high sodium | Serious | Not serious | Serious: proxy outcome and non-usual comparator | Serious | Not assessed | Very low |
| Standard omega-3/fish oil vs. placebo | Serious | Serious: I²=85.9% | Not serious | Serious | Not assessed | Very low |
| Mixed n-3/n-6 LCPUFA vs. placebo | Serious | Not assessable | Not serious | Very serious | Not assessed | Very low |
| PCSO-524 vs. placebo | Not serious | Not assessable | Not serious | Serious: one trial, n=20 | Not assessed; no direct evidence of missing or selectively unreported results | Moderate |
| Lycopene vs. placebo | Serious | Serious: I²=90.4% | Serious: proxy outcome and post hoc subgroup | Very serious: CI includes important benefit and no effect | Not assessed | Very low |
| Vitamin C vs. placebo | Serious | Not assessable | Not serious | Very serious: n=8 | Not assessed | Very low |
| Caffeine 5-6 mg/kg vs. placebo | Serious | Not serious | Not serious | Serious | Not assessed | Low |
| Caffeine 9-10 mg/kg vs. placebo | Serious | Not serious | Not serious | Serious | Not assessed | Low |
| Beta-carotene vs. placebo (responders) | Very serious: post-randomization selection | Not assessable | Serious | Serious | Not assessed | Very low |
| Tangeretin plus whey protein vs. whey protein | Serious | Not assessable | Not serious: additive-effect contrast | Very serious: CI includes important benefit and no effect | Not assessed | Very low |

Note: GRADE was applied to each intervention-comparator-outcome body of evidence. A 5-percentage-point threshold was used only as an operational aid for imprecision and is not a validated minimal clinically important difference. Inconsistency and publication/reporting bias were not assessed when the available evidence did not permit a reliable judgment.

# S8. Secondary outcomes and adverse events

Table S8. Reporting of secondary outcomes and adverse events.

| **Secondary outcome** | **Reporting** | **Synthesis** | **Interpretation** |
| --- | --- | --- | --- |
| EIB incidence/protection | A few studies used different thresholds or responder definitions | Not pooled | Differences in definitions and reporting precluded a reliable summary estimate. |
| Respiratory symptoms | Reported in vitamin C, PCSO-524, and some fish-oil studies | Not pooled | Scales, time points, and reporting methods were inconsistent; a definitive effect could not be established. |
| Rescue-medication use | Reported sporadically in low-sodium, fish-oil, and PCSO-524 studies | Not pooled | Data were insufficient to determine whether nutritional interventions reduce rescue-medication use. |
| Exercise performance | Reported in few studies using different performance measures | Not pooled | Evidence was sparse and heterogeneous. |
| Adverse events | Most studies did not report harms systematically or only stated that treatment was well tolerated | Not estimable | Lack of reporting does not establish absence of harm; no reliable safety conclusion can be drawn. |
| Serious adverse events/withdrawals due to adverse events | No consistent, verifiable reporting | Not estimable | Risk could not be assessed. |
| Adherence | Some studies reported capsule counts, dietary records, or biomarkers | Narrative only | Methods were inconsistent across studies. |

Note: The table summarizes clinically relevant secondary and harm outcomes. Lack of adverse-event reporting was not interpreted as absence of harm.
